# The Emerging Role of Robotics in Pelvic Exenteration Surgery for Locally Advanced Rectal Cancer: A Narrative Review

**DOI:** 10.3390/jcm10071518

**Published:** 2021-04-05

**Authors:** Tou Pin Chang, Aik Yong Chok, Dominic Tan, Ailin Rogers, Shahnawaz Rasheed, Paris Tekkis, Christos Kontovounisios

**Affiliations:** 1 Department of Colorectal Surgery, Royal Marsden Hospital, London SW3 6JJ, UK; t.chang@nhs.net (T.P.C.); Ailin.Rogers@rmh.nhs.uk (A.R.); Shahnawaz.Rasheed@rmh.nhs.uk (S.R.); p.tekkis@imperial.ac.uk (P.T.); 2 Department of Surgery and Cancer, Imperial College, London W2 1NY, UK; chok.aik.yong@singhealth.com.sg (A.Y.C.); dominic.tan.ti.ming@gmail.com (D.T.); 3 Department of Colorectal Surgery, Singapore General Hospital, Singapore 169608, Singapore

**Keywords:** robotic surgery, pelvic exenteration, rectal cancer, multivisceral resection, locally advanced rectal cancer, beyond total mesorectal excision

## Abstract

Pelvic exenteration surgery for locally advanced rectal cancers is a complex and extensive multivisceral operation, which is associated with high perioperative morbidity and mortality rates. Significant technical challenges may arise due to inadequate access, visualisation, and characterisation of tissue planes and critical structures in the spatially constrained pelvis. Over the last two decades, robotic-assisted technologies have facilitated substantial advancements in the minimally invasive approach to total mesorectal excision (TME) for rectal cancers. Here, we review the emerging experience and evidence of robotic assistance in beyond TME multivisceral pelvic exenteration for locally advanced rectal cancers where heightened operative challenges and cumbersome ergonomics are likely to be encountered.

## 1. Introduction

It is well recognised that, for rectal cancers with a clear circumferential resection margin (CRM), defined as > 1 mm from the tumour tissue to the surgical radial margin, undertaking a total mesorectal excision (TME) incorporating the entire mesorectal envelope is usually curative. CRM involvement is widely accepted as a strong independent prognostic factor negatively impacting long-term survival in colorectal cancers [1]. In locally advanced rectal cancer, where the tumour extends beyond the mesorectal envelope, thereby involving the CRM or even direct contiguous invasion of the adjacent organ(s), a multivisceral pelvic exenteration beyond conventional TME surgical planes (i.e., a beyond-TME resection) is required. Approximately 6–10% of all rectal cancers involve the adjacent organs at the time of diagnosis and would benefit from an en-bloc excision of both the tumour and its adjacent organs, i.e., pelvic exenteration surgery [2]. The aim of this article is to provide a comprehensive review on the recent developments in the field of robotic surgery in pelvic exenteration surgery for locally advanced rectal cancers with a specific focus on new innovations and emerging frontiers.

## 2. Methods

A literature search was conducted on MEDLINE (PubMed) and Embase using the following search terms: “robotic surgery” and “rectal cancer”. Case reports, cross-sectional studies, case–control studies, cohort studies, and randomized controlled trials (RCT) were considered for this narrative review. In addition to this, we hand-searched the reference lists of the selected articles and relevant reviews.

## 3. Pelvic Exenteration Surgery

Pelvic exenteration was first described in 1948 by Brunschwig, and its use for colorectal cancer was first described in 1959 by Butcher and Spjut [3]. It has long been associated with a high complication and mortality rate, in part due to the technical difficulties associated with handling several organs within the confined pelvic space [4]. While originally intended as a palliative procedure, the practice of pelvic exenteration has significantly evolved, such that the 5-year overall survival rate of patients undergoing pelvic exenteration for advanced pelvic malignancies now lies between 22 and 66% [2] vs. <5% in non-surgical management options [5]. This improvement of long-term outcomes has largely been attributable to better perioperative care combined with the development of improved surgical techniques, especially in the employment of minimally invasive techniques, namely laparoscopic surgery. Whilst laparoscopic surgery is associated with less intraoperative blood loss, quicker recovery rates, less delayed bowel function, and shorter hospital lengths of stay compared to standard TME surgery [6], the approach encounters technical difficulties due to the complex nature of beyond TME pelvic dissection in multivisceral resections, and there is a compelling need for a more ergonomically and visually enhanced minimally invasive approach.

## 4. Robotics in Pelvic Exenteration Surgery

Nanayakkara et al. described the first robotic-assisted pelvic exenteration surgery for locally advanced rectal cancer in 2014 [7] using the da Vinci^®^ surgical system, and, since then, there has been a steady increase in the number of case reports and series demonstrating its safety and feasibility in multivisceral resections for locally advanced and recurrent rectal cancers worldwide [8,9,10,11,12,13,14] (Table 1). The evidence suggests that several generations of da Vinci robots have been used, with comparable operative time, blood loss, and complete oncological resection margins demonstrated between centres. The conversion rates were low, with just one reported case [12]. The postoperative complication rates were low in most case series but were noted to be higher in larger-volume case series, reflecting the morbid nature of multivisceral pelvic exenterations. Utilising the 13-item Case Report (CARE) checklist for quality appraisal [15], the overall standards of case reports and series reported to date were high despite small but non-negligible omissions on follow-up and outcomes pertaining to R0 status and recurrence rates. Most studies did not report on the patient’s perspectives of their experiences and outcomes after having undergone pioneering surgery in their respective centres.

The operative challenges in the pelvis are centred primarily on the surgeon’s ability to establish satisfactory retraction, light, and space for visualisation of tissue planes for safe dissection. In a spatially constrained pelvis, such as those with high body mass index and narrow pelvic inlet, the handling of tissues with standard laparoscopic instruments for purposeful retraction and for adequate visualisation of beyond-TME planes at depth with neighbouring critical structures is inherently limited. This undoubtedly presents multiple ergonomic difficulties for the surgeon, requiring persistent awkward body postures and prolonged static muscle loading, which, over time, leads to a high prevalence of occupational-related musculoskeletal disorders [16]. In the context of previous radical pelvic surgery and/or irradiated tissues where beyond-TME surgery is often performed, the loss of tissue planes and domains combined with radiation fibrosis renders the operative field more cumbersome even to the experienced eye.

**Figure 1:**
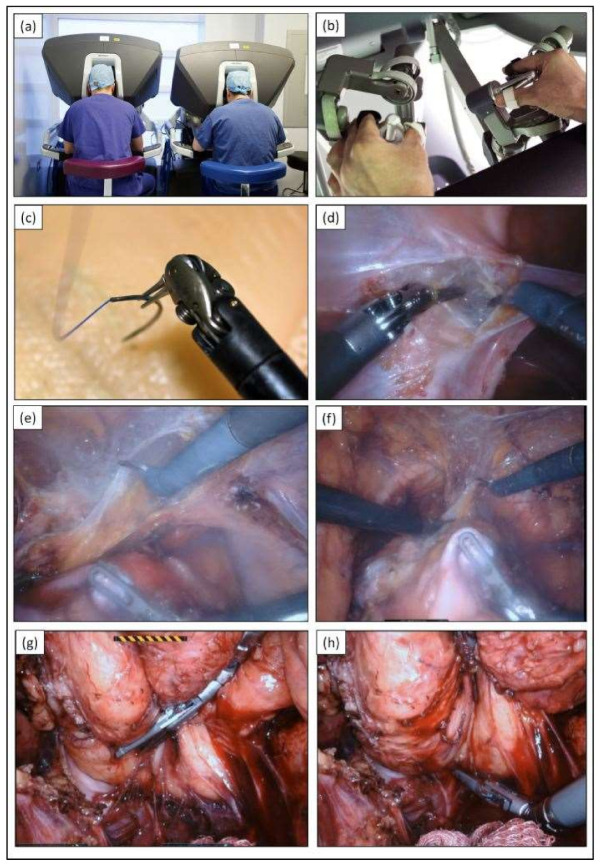
Images of da Vinci ^®^ Surgical System being used in beyond-TME multivisceral pelvic exenteration surgery. (**a**) The da Vinci Xi robot dual console, which allows consultants to supervise trainees during live surgery; (**b**) EndoWrist^®^ technology that allows fully wristed dexterity; (**c**) The articulated ends of robotic instruments; (**d**) Pelvic side wall fascia dissection using the robotic instruments with 3D augmentation; (**e**,**f**) Retropubic mobilisation of the bladder facilitated by its articulated ends; (**g**) Enhanced retraction of the rectum along its posterior wall; (**h**) Utility of the articulated ends in deep pelvic haemostasis.

The robotic-assisted approach may be able to address some of these challenges through its stable operative platform that is integrated with enhanced three-dimensional (3D) visualisation and magnification to augment the surgeon’s depth of perception and clarity of vision in the pelvis, respectively. The operative workflow is delivered through mechatronically enhanced robotic Endo Wrists^®^, which provide enhanced articulation beyond the limits of human wrist movements and eliminates human hand tremors, thus facilitating superior operative dexterity with augmented precision for surgical dissection while preserving stable tissue retraction (Figure 1). The surgeon adopts a sitting posture in the robotic console, with adjustable settings of the viewer’s height and tilt, arm rest height, and the position of the pedal platform, collectively designed and demonstrated to improve the ergonomics of the operating surgeon when compared to laparoscopic surgery [17]. It is worth noting that similar feasibility studies demonstrating the safety and the potential advantages of robotic-assisted pelvic exenteration have also been reported in other tumour groups, such as recurrent endometrial and cervical cancers. Whilst the biology of these tumour groups differs from that of colorectal, the operative challenges remain similar, often in the setting of previous radical pelvic surgery and irradiated tissues with a significant loss of tissue domains, of which encouraging reports of perceived and projected benefits were comparable to those encountered in multivisceral resections for rectal cancers.

At present, the plethora of evidence on robotic-assisted surgery for rectal cancer is centred on case series, non-randomised comparative studies, and a randomised controlled trial comparing robotic assistance against laparoscopic surgery in non-beyond-TME settings, with reported benefits pertaining to less intraoperative blood loss, shorter length of hospital stay, and fewer conversions to open surgery, particularly in males [18]. Concurrent reports have also shown that robotic assistance prolongs the operative time and is more costly than conventional laparoscopic or open approaches, with no major short-term oncological benefits demonstrated so far. The exact role of robotic assistance in standard TME surgery across public health services with escalating time- and cost -constraints remains to be seen. However, it is plausible to hypothesise that appropriately selected cases requiring beyond-TME multivisceral resections, among ergonomically challenging patient subtypes with high BMI and narrow male pelvis, are where the harvested benefits of robotic assistance are likely to be translated into oncological and functional benefits, where considerable improvement is still warranted, therefore substantially justifying its operating time and associated costs. To this end, further prospective studies on safety, efficacy, and operative and short-term oncological outcomes comparing robotic assistance with laparoscopic and open pelvic exenteration surgery constitute the next immediate stage of research priorities.

## 5. Training in Robotic Colorectal Surgery

Over the last decade, it is encouraging to note that there have been reports of the successful integration of robotic colorectal training into residency programmes, notably in the United States, with 95% of its colorectal fellows having received basic robotic training. Elsewhere, Royal College-accredited robotic fellowships were introduced at high-volume centres in London and Portsmouth, United Kingdom, for trainees with robotic subspecialty interests [19], and eight robotic host centres were established through the European Society of Coloproctology to provide operative exposure to standard colorectal procedures for pan-European trainees [20]. The quality of these training opportunities will be further enhanced following the recent development of a European-wide consensus on the standardization of robotic TME, which will provide a structured platform for its competency-based training programme [21]. It is worth noting that most surgeons entering robotic training have had laparoscopic experience performing TME procedures and understanding the planes of dissection, which evidently leads to a shorter learning curve than those seen in the early years of laparoscopic TME adoption [22]. We envisage that a wider acquisition of robotic TME skills and experience with relatively shorter learning curves will allow the more rapid establishment of a larger pool of proficient surgeons equipped with the armamentarium to develop its role and applications in beyond-TME pelvic exenteration surgery.

## 6. Patient Safety Considerations

Given the proximity of dissection to or involvement of critical neurovascular structures in the pelvis, the use of robotic assistance necessitates well-established protocols that enable immediate access to the patient in the event of an iatrogenic event leading to massive haemorrhage or cardiac arrest. The da Vinci^®^ system can be undocked in less than 15 s when required, and emergency protocols for controlled and uncontrolled haemorrhage/cardiac arrest, with specific roles for each member of the operating team, are well established [23,24]. It is, therefore, essential that the whole operating team is familiar with these emergency protocols prior to embarking on robotic pelvic exenteration surgeries. Although over 1.75 million robotic procedures have been performed since 2003, there are low but non-negligible numbers of technical difficulties and complications still being experienced during procedures [25]. Whilst current robotic safety protocols have benefited from the lessons learnt from over a decade’s use in urological, gynaecological, and, more recently, TME surgery, we envisage that further modification of enhanced safety protocols and perioperative support is required in preparation for specific high-risk stages of pelvic exenteration surgeries, such as when bony and vascular planes are entered.

## 7. Limitations and Challenges in Robotic Surgery

However, despite the various technical advantages of robotic surgery, one of its drawbacks is the loss of haptic feedback, which means that the surgeon has to rely heavily on visual cues to steer tissue handling and manipulation. Whilst this is partly circumvented by immersive 3D visualisation, it is evident that pressure, vibration, or shearing forces are not always apparent, and this is occasionally seen during robotic suturing, where unintentional excessive tension applied by the robotic arms results in suture breakage [23]. Despite this limitation, studies to date have demonstrate that robotic-assisted TME surgery has a comparable safety profile to that of laparoscopic approaches [18], and early reports from robotic beyond-TME surgeries reported no significant iatrogenic complications [9,12]. Nevertheless, it is important that future research to elicit any conferred advantages from robotic haptic feedback enhancement takes into account the tactile properties of the multiple tissue planes and visceral interfaces encountered beyond-TME surgery and ascertains whether real-time tactile differentiation of these soft tissue entities confers more safer and complete oncological resections.

The greatest criticism of robotic-assisted surgery is its huge cost, which has hindered its wider usage in healthcare systems across the world. The da Vinci^®^ system comes with a high capital cost, ranging from GBP 1.5 m to GBP 2 m, with annual maintenance costs and additional robotic instruments costs to be considered. This is further compounded by its longer operating time, but, in the context of beyond TME surgery, the robotic docking time constitutes a relatively small proportion of the overall long operating time; therefore, its direct cost consequences should be insignificant. Based on the data from the ROLARR trial, robotic-assisted TME is projected to be at least GBP 1000 more costly than laparoscopic TME surgery [18]. However, in pelvic exenteration surgery, where there are higher average lengths of stay and complication and readmission rates, there is considerable room for improvement. If robotic assistance can be shown to negate some of these aspects, significant cost savings could potentially be made.

## 8. Future Directions

Moving forward, evidently, more studies are required to generate robust data linking the delicate interplay between perioperative factors to the overall cost of care and oncological outcome factors that underpin the establishment of its role in beyond-TME multivisceral pelvic exenteration surgery. Recent promising results from the utilisation of other new robotic platforms in colorectal cancer resections, such as the Versius^®^ surgical system [26], may promote a more competitively priced market for wider usage across healthcare systems. The early evidence so far suggests that the perioperative outcomes are comparable across different centres, although high complication rates have been noted in larger case series, perhaps reflecting the true cohort of the complex nature of multivisceral exenteration surgery. Nevertheless, it can be performed without compromising the quality of oncological resection status.

It is worth noting that the current robotic platforms have introduced windows of opportunity for the integration of new medical imaging techniques such as fluorescence-guided surgery, real-time 3D modelling, and stereotactic navigation that represent promising avenues for improving the precision and accuracy of surgical dissection to improve the completeness of resection margins. In preliminary studies, indocyanine green, for example, has emerged as a promising tool for the real-time intraoperative assessment of tissue perfusion and the detection of sentinel nodes during robotic pelvic dissection in rectal cancer surgery [27]. Recent advances in software segmentation of MRI images have facilitated 3D intraoperative modelling to produce true-size, detailed representation of organs, soft tissues, and critical structures that could potentially distinguish fibrotic from diseased tissues following chemoradiotherapy treatment [28,29,30]. Additionally, the integration of real-time stereotactic navigation to assist in the identification of critical structures in the pelvis has been shown to be feasible on a robotic platform [31]. These advancements, albeit preliminary at this stage, are potential harbingers of a new era in robotic surgery, where computer-assisted systems are fused with established robotic platforms to facilitate better decision-making intraoperatively. Noting the challenges faced in its diffusion of uptake in TME surgery, we envisage that the true niche of robotic assistance lies in applications whereby the ergonomic and visual enhancement of the operating field and improvement of postoperative outcomes are significantly warranted, such as multivisceral pelvic exenteration surgery.

Another aspect that warrants prudent investment of attention would be to encourage patient engagement and involvement in the design and conduct of future clinical studies on robotic pelvic exenteration surgery. This may help to resolve any discrepancies between existing public perceptions of robotic surgery and the clinical evidence [32]. As stakeholders in their personalised care, this approach may potentially capture patient’s perceptions of factors that support and constrain the integration of robot-assisted surgery into routine practice as we transition towards the healthcare systems of the future, which are increasingly patient-driven.

## Figures and Tables

**Table 1:** Original studies utilising da Vinci^®^ Surgical System for multivisceral pelvic exenteration surgery for locally advanced including recurrent rectal cancers.

Author, Year, Country	*n*	Robot Generation	Type of Surgery	Mean Operating Time (hours)	Mean Blood Loss (mL)	Perioperative Complications	Mean ITU Stay (days)	Mean Length of Stay (days)	Resection Margin Status (R0)	Recurrence Rates	CARE Score, Incomplete Items
Williams et al., 2021 Australia [8]	5	Si, Xi, S	Robotic APER, cystoprostatectomy, ileal conduit formation (*n* = 2); robotic APER and prostatectomy (*n* = 2); robotic ultralow AR colorectal anastomosis, prostatectomy, and loop ileostomy (*n* = 1)	7.8 (3–11)	520 (150–1000)	Mortality (*n* = 1). Small bowel obstruction and pneumonitis at 31 days post-op.	1 (1–1)	9 (6–34)	4/5 (80%)	2/5 (40%) at 21 and 24 months	12/13, IPP
Smith et al., 2020, Australia [9]	8	Xi (*n* = 4) Si (*n* = 4)	Robotic APER and prostatectomy (*n* = 3); robotic ultralow AR, TAHBSO, and partial vaginectomy (*n* = 2), robotic APER, cystoprostatectomy, ileal conduit formation (*n* = 1); robotic ultralow AR, colorectal anastomosis, cystoprostatectomy, ileal conduit, and loop ileostomy (*n* = 1); robotic low AR and prostatectomy (*n* = 1)	8.5 (6–10)	6 out of 8 did not receive perioperative transfusion; 2 out of 8 received 2 units	None	1 (0–3)	15 (7 to 26)	8/8 (100%)	Disease-free at 12 months	12/13, IPP
Heah et al., 2020 Singapore [10]	3	S	Robotic APER with en-bloc prostatectomy with vesico-urethral Anastomosis (*n* = 1); robotic ultra-low anterior resection with J-pouch coloanal anastomosis and en-bloc prostatectomy with vesico-urethral anastomosis (*n* = 1); robotic anterior resection with en-bloc prostatectomy and defunctioning ileostomy (*n* = 1)	N/A	700 (600–800)	None	N/A	12.6	2/3 (67%)	N/A	12/13, FUO
Raj Kumar et al., 2020 [11] India	1	Si	Robotic APER and prostatectomy	9	750	None	N/A	N/A	1/1 (100%)	Disease-free at 6 months	11/13, IPP and FUO
Shin et al., 2016 USA [12]	22	N/A	Robotic TME, inferior mesenteric, en bloc resection of prostate or periprostatic tissue (seminal vesicle, vas deference) (*n* = 8); vaginal wall (*n* = 5); small bowel (*n* = 3); bladder wall or pericystic soft tissue (*n* = 3); coccyx (*n* = 2); appendix (*n* = 2); uterus (*n* = 1)	7 (5.5–8.5) *	225 (150–350) *	12/22 (52%) > 1 complication (*n* = 4); Pelvic abscess (*n* = 4); Wound infection (*n* = 3); Wound dehiscence (*n* = 1); UTI (*n* = 2); Haemorrhage (*n* = 1); Urinary retention (*n* = 3); Urinary leak (*n* = 1); Ileus (*n* = 5). Re-admissions (*n* = 6); Re-operations (*n* = 3)	N/A	4.5 (4–6) *	22/22 (100%)	N/A for exenteration group	12/13, IPP
Winters et al., 2015, USA [13]	1	Si	Robotic APER and cystoprostatectomy	9.5	350	None	1	7	N/A	N/A	11/13, IPP and FUO
Shin et al., 2013, South Korea [14]	3	N/A	Robotic ultralow AR, coloanal anastomosis, right hemicolectomy, prostatectomy, loop ileostomy (*n* = 1); Robotic APER, partial cystoprostatectomy, vesico-urethral anastomosis (*n* = 1); Robotic APER, cystoproatatectomy, ileal conduit formation (*n* = 1)	8.9 (8–9.7)	530 (300–700)	Vesico-urethral anastomotic leak at day 14 post-op	N/A	18 (8–28)	2/3 (66%)	Disease at 14 months (*n* = 1), N/A (*n* = 2)	12/13, IPP
Nanayakkara et al., 2014 UK [7]	1	N/A	Robotic APER, TAHBSO, posterior vaginectomy	N/A	N/A	None	N/A	8	1/1 (100%)	N/A	11/13, IPP and FUO

ITU: Intensive care unit, AR: Anterior resection, APER: Abdominoperineal excision of the rectum, TAHBSO: Total abdominal hysterectomy bilateral salpingoophorectomy, TME: Total mesorectal excision, UTI: Urinary tract infection, CARE checklist: CAse REport Statement and Checklist, IPP: Involvement of patient’s perspective, FUO: Follow-up and outcomes, * Data presented as median and interquartile range.
